# Efficacy of Platelet-Rich Plasma in Reduction of Post-Operative Split-Thickness Skin Graft Loss and Hematoma Formation: A Meta-Analysis

**DOI:** 10.7759/cureus.15160

**Published:** 2021-05-21

**Authors:** Anshika Tyagi, Apurv Gupta, Vicente I Martires III, Moises Romo, Ishan Garg, Diego Tapia, Paola Gudino, Sinyun Lam

**Affiliations:** 1 Department of Plastic Surgery, Maulana Azad Medical College, New Delhi, IND; 2 Department of Surgery, University of Santo Tomas Faculty of Medicine and Surgery, Manila, PHL; 3 Department of Medicine and Nutrition, University of Guanajuato, Leon, MEX; 4 Department of Medicine, Ross University School of Medicine, Bridgetown, BRB; 5 Faculty of Medicine, Pontifical Catholic University of Ecuador, Quito, ECU; 6 Department of Family Medicine, Aviva Health, Roseburg, USA

**Keywords:** split-thickness skin graft, graft recipient bed preparation, wound reconstruction, wound repair, platelet-rich plasma/prp

## Abstract

Split-thickness skin grafting is a very popular technique of wound closure, especially for large wounds. The success rate of a split-thickness skin graft (STSG) has consistently been in the range of 70-90%. Multiple techniques have been introduced to further improve graft survival, for example, the use of autologous platelet-rich plasma (PRP), thrombin gel, platelet-rich fibrin matrix, and negative pressure wound therapy. We evaluated the impact of PRP use on the survival of STSG through a meta-analysis. We conducted the analysis in accordance with Preferred Reporting Items for Systematic Review and Meta-Analyses (PRISMA) protocol and performed a literature search using the following databases: PubMed, Cochrane, and ClinicalTrials.gov. A total of 126 articles were yielded by the search, out of which four randomized controlled trials (RCTs) were included according to our eligibility criteria and irrelevant articles were excluded. The intervention group received PRP application to the wound bed before applying the graft while the control group received treatment with conventional fixation procedures (sutures and staples). We estimated the pooled odds ratio with a 95% confidence interval (CI). The total number of participants in the analysis was 460. The participants had wounds of varying etiology. Post-operative graft loss and hematoma formation were taken to be the primary and secondary outcome measures, respectively. Thirty-four participants suffered graft loss of differing extent in the control group while three participants suffered graft loss in the intervention group. The pooled odds ratio for graft loss was 0.15 (95% CI: 0.05-0.49; I^2^=38%; p=0.184) signifying that PRP use decreased the odds of graft loss by 85%. For our secondary outcome measure, 440 participants were studied. Forty-four participants suffered hematoma formation in the control group versus 11 in the intervention group. The pooled odds ratio for hematoma formation was calculated as 0.21 (95% CI: 0.09-0.50; I^2^=0%; p=0.869) signifying that PRP use decreased the odds of hematoma formation by 79%. PRP appears to significantly impact graft survival, and further studies are needed to strengthen the evidence base for its use in split-thickness skin grafting.

## Introduction and background

Split-thickness skin grafting is a technique where cutaneous tissue is transferred from one region of the body to the other. Split-thickness skin graft (STSG) consists of a full epidermis and partial dermis harvested from a donor site which then heals on its own. STSGs are most often used to cover large defects because their donor sites are able to heal better. A portion of the dermis that is left behind after harvest of STSG helps in the regrowth of new skin at the donor site [1].

Since grafts do not contain a blood supply of their own, the wound bed that they are applied on should be clean, healthy, and well-vascularized to improve the graft take. Several studies have reported the split-thickness graft takes to be around 70-90%. A number of ways have been tried to improve the take, for example, bolstering with the help of negative pressure wound therapy, hypertonic glucose with negative pressure wound therapy, antimicrobial dressing combined with negative pressure wound therapy, and autologous platelet-rich plasma (PRP) [2-4].

Several authors have reported using PRP to prepare a wound bed before placing the graft. To completely establish the role of PRP in preventing graft loss, it was felt that there is a need to perform a meta-analysis of studies exploring the benefits of PRP in STSG application.

## Review

Data source and searches

**Figure 1:**
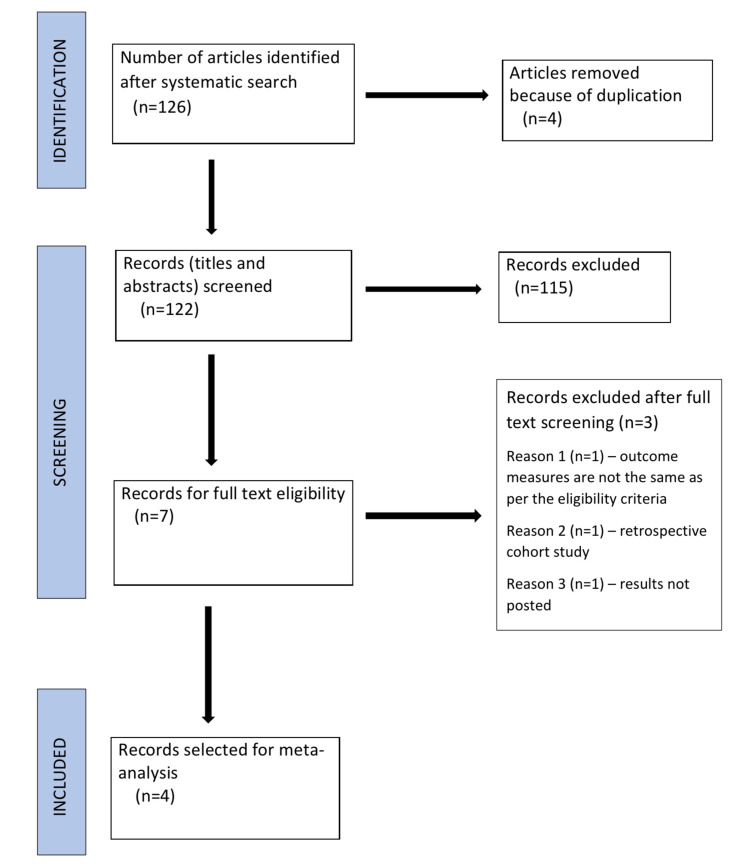
PRISMA flow diagram

The meta-analysis was conducted according to the Preferred Reporting Items for Systematic Review and Meta-Analyses (PRISMA) protocol (Figure 1). We carried out a systematic electronic search using the following databases: PubMed, Cochrane, and ClinicalTrials.gov from the date of inception of each database to April 2021. The following search terms with Boolean operators were used: platelet-rich plasma AND split-thickness skin graft, platelet-rich plasma AND wound bed preparation, split-thickness skin graft AND wound bed preparation. The reference lists of all eligible studies were also checked for any additional relevant articles.

Study selection and eligibility criteria

The search led to the identification of 126 articles. The titles and abstracts of the articles identified by the search were independently reviewed by two authors against the eligibility criteria (Table 1). Any disagreement on the eligibility of titles was resolved by a third independent reviewer. This was followed by retrieval of full texts of eligible titles and abstracts. Review articles, case reports, abstracts, and animal studies were excluded.

**Table 1:** Eligibility criteria RCT: randomized controlled trial, PRP: platelet-rich plasma.

	Inclusion	Exclusion
Population	Adult and pediatric patients with wounds of varying etiology	None
Intervention	PRP applied on wound bed before grafting	PRP application combined with any other intervention
Comparators	Not applicable	Not applicable
Outcome measures	Post-operative graft loss and hematoma formation	None
Study design	Peer-reviewed English language RCTs	Observational studies, abstracts, review articles, animal studies, and case reports

Data extraction and quality evaluation

A data extraction sheet was developed for the purpose of recording the following information from the included studies: author, year of publication, design of the study, number of patients enrolled, distribution of intervention and control group, primary and secondary outcome measures for intervention as well as control group (Table 2). Joanna Briggs Critical Appraisal Checklist for Randomised Controlled Trials was used to assess the quality of the included studies (Table 3). Publication and small study biases were assessed through the generation of a funnel plot and assessment for asymmetry.

**Table 2:** Data extraction sheet RCT: randomized controlled trial, NR: not reported. ^a^Data reported for post-operative day 7.

Study	Year of publication	Design of study	Total number of patients (n)	Intervention group (n)	Control group (n)	Graft loss in the control group (n)	Graft loss extent in the control group (%)	Graft loss in the intervention group (n)	Graft loss extent in the intervention group (%)	Hematoma formation in the control group (n)	Hematoma formation in the intervention group (n)
Waiker and Shivalingappa [5]	2015	RCT	200	100	100	15	NR	0	0	15	4
Sonker et al. [6]	2015	RCT	20	20	20	11	50-100	0	0	NR	NR
Dhua et al. [7]	2019	RCT	40	20	20	2^a^	0-35	1^a^	0-10	6^a^	1^a^
Gupta et al. [8]	2020	RCT	200	100	100	6	40-70	2	50	8	2

**Table 3:** Joanna Briggs Institute Critical Appraisal Checklist for randomized controlled trials

Criterion	Waiker and Shivalingappa [5]	Sonker et al. [6]	Dhua et al. [7]	Gupta et al. [8]
Was true randomization used for the assignment of participants to treatment groups?	Yes	Yes	Yes	Yes
Was allocation to treatment groups concealed?	Yes	Yes	Yes	Yes
Were treatment groups similar at the baseline?	No	No	No	Yes
Were participants blind to treatment assignment?	Yes	Yes	Yes	Yes
Were those delivering treatment blind to treatment assignment?	No	No	No	No
Were outcome assessors blind to treatment assignment?	No	No	No	No
Were treatment groups treated identically other than the intervention of interest?	Yes	Yes	Yes	Yes
Was follow-up complete and if not, were differences between groups in terms of their follow-up adequately described and analyzed?	Yes	Yes	Yes	Yes
Were participants analyzed in the groups to which they were randomized?	No	No	No	No
Were outcomes measured in a reliable way?	Yes	Yes	Yes	Yes
Was appropriate statistical analysis used?	Yes	Yes	Yes	Yes
Was the trial design appropriate, and any deviations from the standard RCT design accounted for in the conduct and analysis of the trial?	Yes	Yes	Yes	Yes

Statistical analysis

A forest plot was used to illustrate the analysis of the data. We evaluated heterogeneity among studies using the Q statistics and I^2^ index, assuming that I^2^ values of 25%, 50%, and 75% represent low, medium, and high heterogeneity, respectively. We considered an I^2^ value of greater than 50% as indicative of substantial heterogeneity. Substantial heterogeneity in our data was not observed. The pooled estimate was calculated based on the fixed-effects model because of low heterogeneity and was reported using Woolf’s (inverse variance) method. For the detection of publication bias, we used direct observation of funnel plot symmetry, Egger’s regression asymmetry test, and Begg’s rank correlation methods. In the evaluation of publication bias using the Egger’s and Begg’s tests, P<0.05 is considered to be statistically significant. Funnel plots were used for the assessment of publication bias by graphical inspection.

RStudio version 1.0.136 (RStudio, Inc., Boston, MA) and “Meta” R package (version 4.9-7, The R Foundation, Vienna, Austria) were used for the meta-analysis.

Results

**Figure 2:**
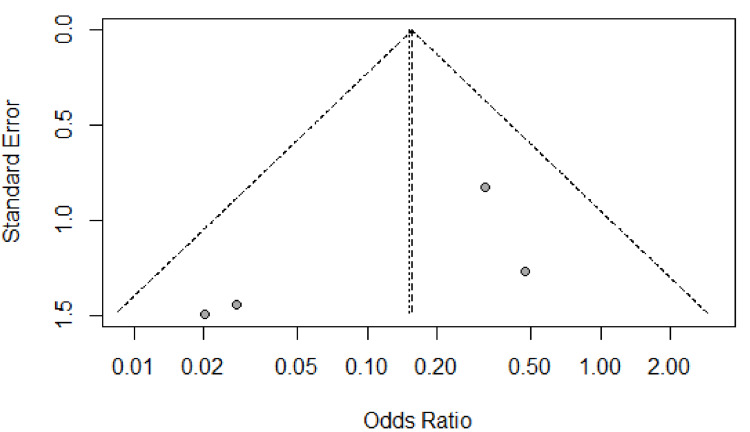
Funnel plot for potential publication bias of studies with regards to primary outcome measure of post-operative graft loss

**Figure 3:**
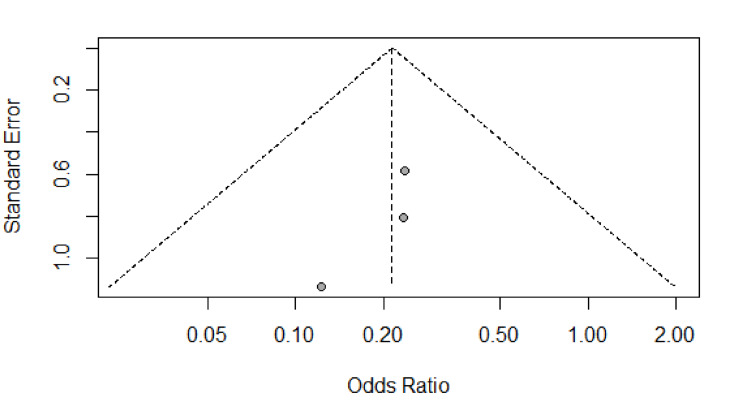
Funnel plot for potential publication bias of studies with regards to secondary outcome measure of hematoma formation

Publication and small-study biases were assessed through the generation of a funnel plot and assessment for asymmetry. Egger’s linear regression test and Begg’s correlation test were used to investigate any suspected asymmetry. From the overall result of the meta-analysis, the central estimate was plotted (the vertical dashed line), and the 95% confidence intervals (CI) were drawn (the diagonal dashed lines) to form the funnel or inverted V. If there was any publication bias, then the studies would not be equally distributed within the inverted V (Figures 2 and 3).

Egger’s test and Begg’s correlation test were used to detect potential publication bias (P>0.05 was considered representative of no statistically significant publication bias). There was no considerable publication bias as detected by Egger’s tests and Begg’s correlation test.

Graft Loss

**Figure 4:**
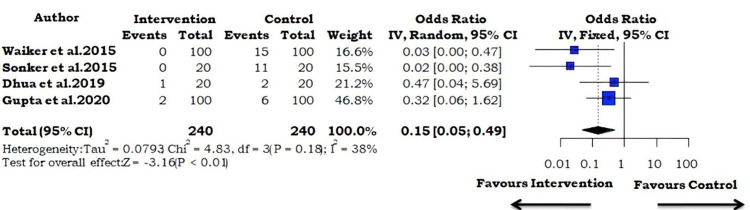
Forest plot using the fixed-effect model for the outcome measure of post-operative graft loss Horizontal bars indicate the amount of variation (95% confidence intervals of the parameter estimates). Sizes of squares indicate the weight allotted to individual studies in the pooled effect size [5-8].

The results of our meta-analysis of four studies indicate a pooled odds ratio of 0.15 (95% CI: 0.05-0.49; I^2^=38%; p=0.184; Figure 4). Thus, it can be concluded that PRP use decreases the odds of graft loss by 85%.

Hematoma Formation

**Figure 5:**
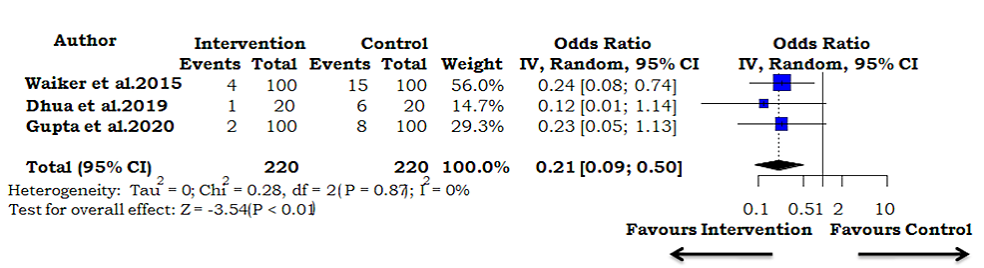
Forest plot using the fixed-effect model for the outcome measure of hematoma formation Horizontal bars indicate the amount of variation (95% confidence intervals of the parameter estimates). Sizes of squares indicate the weight allotted to individual studies in the pooled effect size [5,7,8].

The results of our meta-analysis of three studies indicated a pooled odds ratio of 0.21 (95% CI: 0.09-0.50; I^2^=0%; p=0.869; Figure 5). They demonstrated that the use of PRP decreased the odds of post-operative hematoma formation by 79%.

A summary of the analysis is presented in Table 4.

**Table 4:** Summary of statistical analysis CI: confidence interval.

Outcome	Model fitted	Pooled odds ratio	95% CI	Z-value	Fixed-effect P-value	I^2^	Test of heterogeneity P-value	Egger’s linear regression test of funnel plot asymmetry	Begg’s linear regression test of funnel plot asymmetry
Graft loss	Fixed-effect model	0.15	0.05–0.49	−2.83	0.0013	38%	0.1845	0.258	0.041
Hematoma formation	Fixed-effect model	0.21	0.09–0.50	−3.54	0.0004	0.0%	0.8691	0.325	0.117

Discussion

There are a lot of complications associated with STSG. Failure of graft adherence to the wound bed after application of graft, edema, and hematoma formation which prevent the adherence of graft to the wound bed, contracture, and scar hypertrophy are the more common ones [1]. The aim of this study was to shed some more light on the adjunct role of PRP in split-thickness skin grafting. PRP, by definition, contains a platelet concentration of a minimum of 10,00,000 platelets/µl in 5 ml of plasma ​​​​but it is more than just platelets [5]. It also contains many growth factors which are conducive to various anabolic pathways like angiogenesis [9].

The methods that were used to prepare PRP were different among the studies. Two studies used a single spin procedure [5,8]. One study used a double spin procedure [7]. One study carried out apheresis using the cell separation method [6]. The intervention was defined as the application of PRP to the wound bed prior to the application of graft. Three studies compared graft fixation using conventional methods of sutures and staples with graft fixation using the application of PRP alone on the wound bed [5,7,8]. One study used one-half of the wound as control with fixation of STSG by sutures and staples while the other half of the same wound was treated with PRP alone before graft fixation [6].

The outcome measures chosen for our study were post-operative graft loss (reported in all four included studies) and hematoma formation (reported in three of the included studies). The study by Dhua et al. reported post-operative graft loss and hematoma formation in a graphical representation in which data were recorded for post-operative day 7, day 14, and day 21 [7]. We took into account the data for day 7 in our study because it was felt that data for days 14 and 21 reflect the effects of other variables not within the scope of our study.

According to our analysis, it appears that the addition of PRP to wound bed before application of STSG reduces the rate of graft loss and hematoma formation. However, our study is not without its limitations. First of all, an insufficient number of studies have been carried out addressing the topic of concern. Furthermore, the authors have only taken into consideration the studies in the English language. Moreover, there were differences with regard to methods of PRP preparation among the studies. In order to clearly distinguish the role of PRP in split-thickness skin grafting, further high-quality RCTs should be performed. It is recommended that a uniform method of PRP production be used in RCTs on this topic along with the recruitment of a patient population with similar wound etiology. These considerations will make comparison among studies more reliable.

Recent years have also seen the development of a new generation of platelet concentrates, for example, platelet-rich fibrin matrix [10], platelet-protein film [11], and fibrin glue [12]. They have shown promising results in skin graft healing but they are still in development stages and the evidence base for their use in routine practice is not very strong.

## Conclusions

STSGs are one of the many important ways of treating chronic wounds. It has long been desirable to find appropriate modifications to improve its survival. In this study, we analyzed the data available to investigate the benefits of PRP in split-thickness skin grafting. Our study considered two outcome measures: post-operative graft loss and hematoma formation. The use of PRP decreased the odds of graft loss by 85% and the odds of hematoma formation decreased by 79%. Thus, it can be concluded that PRP improves outcomes for STSGs. The authors acknowledge that a limited number of RCTs have been conducted addressing the research question and further studies should be carried out to improve the quality of additional meta-analyses on this topic.
